# Molecular Genetics of Pigment Dispersion Syndrome and Pigmentary Glaucoma: New Insights into Mechanisms

**DOI:** 10.1155/2018/5926906

**Published:** 2018-03-26

**Authors:** Adrian A. Lahola-Chomiak, Michael A. Walter

**Affiliations:** Department of Medical Genetics, Faculty of Medicine, University of Alberta, 8-32 Medical Science Building, Edmonton, AB, Canada

## Abstract

We explore the ideas and advances surrounding the genetic basis of pigment dispersion syndrome (PDS) and pigmentary glaucoma (PG). As PG is the leading cause of nontraumatic blindness in young adults and current tailored interventions have proven ineffective, a better understanding of the underlying causes of PDS, PG, and their relationship is essential. Despite PDS being a subclinical disease, a large proportion of patients progress to PG with associated vision loss. Decades of research have supported a genetic component both for PDS and conversion to PG. We review the body of evidence supporting a genetic basis in humans and animal models and reevaluate classical mechanisms of PDS/PG considering this new evidence.

## 1. Introduction

Pigment dispersion syndrome (PDS) is the shedding of pigment from the posterior surface of the iris into the anterior segment following the flow of aqueous humour. This shedding does not independently impair vision in most affected individuals. However, a subset of patients with PDS progresses to pigmentary glaucoma (PG) with high intraocular pressure (IOP) and glaucomatous optic neuropathy. To date, although several population studies have established a relationship between these two disorders, the underlying pathology remains cryptic. A heterogeneous and possibly complex genetic component appears to underlie at least a proportion of PDS/PG cases. Understanding this genetic component can not only provide insight into the underlying pathology of PDS/PG but also form the basis for rationally designed therapeutics for this important cause of blindness worldwide.

## 2. Pathophysiology

The defining characteristic of PDS is the bilateral shedding of pigment from the posterior iris pigment epithelium (IPE) and the subsequent deposition of this pigment in the anterior segment, first described in 1899 [1]. Pigment lost in this way can be visualized gonioscopicly as iris transillumination defects which describe depigmented zones that abnormally allow light to pass through them [2]. These slit-like depigmented zones tend to be observed radially in the midperipheral iris and, according to ultrasound biomicroscopy studies, patients with PDS often have abnormal iridozonular contacts [3, 4]. As described in more detail below, these abnormal contacts have previously been proposed to be responsible for pigment shedding via a mechanical rubbing model [3]. Iris transillumination defects are observed in approximately 86% of patients with PDS [5]. Liberated pigment is transported into the anterior segment via aqueous humour flow. Aqueous humour convection currents are driven primarily by blinking [6] then deposit this pigment in a vertical stripe on the cornea known as a Krukenberg's spindle [1, 7]. This phenotype is observed in around 90% of PDS patients and does not correlate with differences in corneal thickness or density [8, 9]. Possibly, the most important clinical sign in the pathophysiology of PDS is the observation of dense trabecular meshwork pigmentation [7]. PDS patients tend to have a diffuse and uniformly dense pigmented trabecular meshwork, unlike patients with the phenotypically related pseudoexfoliation syndrome where punctate deposits of material in the trabecular meshwork are observed [10].

Histologic examination has revealed that pigment granules are phagocytosed by both corneal epithelial cells and trabecular meshwork cells rather than being adsorbed onto their surface [11–14]. Phagocytic stress causes alterations to the trabecular meshwork extracellular matrix structure and adhesion [15, 16] which could explain the trabecular meshwork dysfunction observed in PDS/PG patients [14]. In PDS/PG patients, trabecular meshwork cells die and exhibit localized necrosis [17]. The resultant reduced conventional aqueous humour outflow which is likely the primary mechanism in the conversion from PDS to PG as reduced outflow is an established mechanism for IOP increase and glaucomatous optic neuropathy [17, 18]. Nevertheless, the degree of trabecular meshwork pigmentation does not directly correlate with conversion risk but is however related to the severity of optic neuropathy in PG patients [19, 20]. The lens and iris have been suggested to function together in a ball-valve pressure mechanism, called the reverse pupillary block, maintaining one-way aqueous humour flow [21]. Elevated anterior segment pressure may bend the iris posteriorly, increasing iridozonular contact and as a result exacerbate pigment shedding [22, 23]. However, iris bending cannot be solely due to pressure as a study using ex vivo iris explants showed that iris bowing is a normal feature of iris dilator muscle activity and position [24]. Hyperplastic iris dilator muscles have been observed in several patients with PDS, and this dysfunction may contribute to posterior iris bowing [25–27].

Although currently limited, some evidence has accrued to support the involvement of the retinal pigment epithelium (RPE) in PDS/PG. Patients with PDS have significantly lower Arden ratios than patients with primary open-angle glaucoma (POAG) or ocular hypertension (OHT) which may indicate RPE degeneration [28]. Lattice retinal degeneration occurs in 22–33% of PDS/PG cases which is high, despite the known association between PDS and myopia [29–31]. An estimated 12% of eyes with PDS also experience retinal detachment, occurring in 5.5-6.6% of total PDS cases [22, 31, 32]. Together these data support a more general involvement of pigmented cells in the pathology of PDS/PG, but further characterization of possible RPE dysfunction associated with PDS/PG is necessary.

## 3. Epidemiology

At a population scale, there are three main questions to answer about PDS and PG. As PDS is the underlying condition, it is important to know how many people are affected as well as details regarding their demographic characteristics. The incidence of PDS has been estimated to be between 1.4 per 100,000 to 4.8 per 100,000^5^ in the United States. However, some estimates place prevalence as high as 2.45% in the United States [33]. Screening for PDS however is complicated by its subclinical nature, the fact that pigment dispersion is more easily observed in lightly pigmented eyes and the phenomenon of symptom abatement known as “burn-out” [5, 20]. People affected by PDS may not seek out eye exams since their vision is not impaired, and affected individuals may be asymptomatic for obvious pigmentary defects due to “burn-out,” together leading to an underestimation of PDS prevalence. Burn-out typically occurs in older individuals and it is possible some patients who present with glaucomatous optic neuropathy, who are then diagnosed with POAG, may be more accurately described as PG cases with burn-out. PDS is known to affect young myopes which may explain some of the structural iris pathologies associated with the disease [7, 31, 34]. North American studies have established a higher prevalence of PDS in white patients and a lower than expected incidence of PDS in black patients [34–36], but the aforementioned ability to more readily detect aberrantly located pigment in light-coloured eyes might lead to an ascertainment bias.

Conversion from PDS to PG is a highly variable and heterogeneous phenomenon impacted by both genetic and environmental factors. For example, despite the prevalence of PDS being approximately equal in both men and women, more males progress to PG [6, 19, 22, 31] and that conversion occurs about a decade earlier in men than women [6, 21, 31, 34, 37]. PDS patients have an increased family history (4–21%) of glaucoma [5, 31, 38]; however, that percentage increases greatly in patients with PG (26–48%) [5, 34, 39, 40]. Rigorous exercise has been shown to induce pigment dispersion, enhance posterior iris bending, and increase IOP which all contribute to conversion risk [41–44]. IOP is a major risk factor for PDS to PG conversion, with the increase in risk being proportional to the increase in IOP [5, 45]. The actual rate of conversion is highly variable between studies and seems to in part depend on the ethnic background of patients. Conversion rates as high as 35–50% have been reported in US populations [19, 37, 46]. However, in another study which evaluated conversion over time, the conversion rate was estimated at 10% at 5 years and 15% at 15 years [5]. In a Latin American cohort, the conversion rate was observed to be 37.5% at 50 months which is in good agreement with US studies [45]. However, in a Pakistani cohort, the observed conversion rate was only 4% at 15 years which may support ethnicity as a risk factor for conversion [47]. Ultimately, the diversity in conversion rates supports the observation of heterogeneous genetic and environmental risk factors. In the Western world, PG represents 1–1.5% of total glaucoma cases and, due to its early age of onset, is the most common cause of nontraumatic glaucoma in young adults [33, 48] making it an important cause of debilitating blindness.

## 4. Human Genetics

Current research on the genetic component of PDS/PG supports a genetically heterogeneous and possibly complex inheritance model. Analysis of four 3-generation pedigrees with Irish or mixed Western European ancestry affected by PDS/PG supported an autosomal dominant mode of inheritance given the identification of affected individuals in every generation without a sex bias [49]. Using microsatellite markers, a chromosomal region named *GPDS1* (glaucoma-related pigment dispersion syndrome 1) (OMIM ID 600510) was mapped to the human chromosome 7 (7q35-q36) in a subset of patients. To date, this linkage has not been replicated by other mapping studies. Additionally, no candidate genes in this region have been successfully associated with PDS/PG. Of several genes in the region, the most promising candidate is likely human endothelial nitric oxide synthase (*NOS3*) as it is known to play a role in maintaining vascular tone and dysfunction and may contribute to structural abnormalities of the iris [50–52]. However, mutations in *NOS3* have not been reported to be associated with PDS/PG to date.

Another region, on chromosome 18, has also been associated with PDS/PG in several studies. Using a single pedigree, significant linkage to the 18q11-q21 region was observed [53] and later analysis of four additional pedigrees not linked to *GPDS1* found significant linkage to the 18q21 region, assuming an autosomal dominant mode of inheritance [54]. Finally, there exists one case study of an Estonian man with PDS harbouring novel deletions on both the nearby 18q22 and 2q22.1 [55]. However, as for the *GPDS1* locus, no genes in these regions have been associated with PDS/PG.

Two candidate genes associated more broadly with other subtypes of glaucoma, myocilin (*MYOC*), and lysyl oxidase homolog 1 (*LOXL1*) have shown limited association with PDS/PG. *MYOC* is well known for its association with several subtypes of glaucoma including juvenile open-angle glaucoma (JOAG) and primary open-angle glaucoma (POAG) [56–58]. Several cases of potentially damaging mutations in *MYOC* in patients with PDS/PG have been observed [59–61], and *MYOC* is expressed in several ocular tissues including the iris which makes it biologically plausible, despite its still cryptic biological function. However, the very small number of *MYOC* variants found associated with PDS/PG suggests that MYOC is either a very infrequent cause of PDS/PG or that this association is spurious. Given the phenotypic similarities between pseudoexfoliation syndrome (PXS) and PDS (deposition of material in the anterior segment), several studies have investigated a possible association between *LOXL1*, a gene strongly associated with PXS [62–66], and PDS/PG. To date, no causal association of PDS/PG and LOXL1 variants has been observed [67, 68] but variants in LOXL1 could act as a modifier of both of disease risk and age of onset [67, 69]. Interestingly, a patient with coexisting [70] PXS and PDS has been described, supporting again the idea that these related disorders are separate clinical and genetic entities.

There may be also some overlap between PDS and the rare recessive disease Knobloch syndrome (OMIM number 267750) caused by mutations in *COL18A1* [71, 72]. Knobloch syndrome is a developmental disorder with ocular abnormalities and severe skull formation defects. Recently, it is has been reported that PDS and PG are a hallmark sign of Knobloch syndrome and that understanding PDS/PG is important for management of Knobloch syndrome [73]. However, given the severity of the other diagnostic symptoms of Knobloch syndrome, variants in *COL18A1* are unlikely to cause a large proportion of PDS/PG cases. Two case reports have associated Marfan syndrome (OMIM number 154700) with PDS/PG and suggested that *FBN1* variants, while not causative for PDS, may contribute to conversion to glaucoma [74, 75]. Although glaucoma generally has been associated with Marfan syndrome [76], there currently exists insufficient evidence to associate PDS/PG directly with Marfan syndrome or variants in *FBN1*.

## 5. Animal Studies

Whereas human studies have failed to elucidate any gene associated with PDS/PG, animal research has successfully identified several genes associated with similar phenotypes. Undoubtedly, the most significant progress has been made using the DBA/2J mouse glaucoma model which has proven invaluable to both PDS/PG research and understanding of glaucomatous optic neuropathy as a whole [77–81]. DBA/2J mice were observed to sporadically develop iris atrophy, pigment dispersion, increased IOP, and glaucoma-like retinal ganglion cell death [81]. Later, the genes responsible for these sporadic phenotypes were mapped to two main genes: *Tryp1* and *Gpnmb* which accounted for the iris atrophy and pigment dispersion respectively [77]. Iris pigment dispersion and associated atrophy have also been observed in several other mouse models and causative genes together implicate melanosome genes as playing a central role in iris pigment dispersion pathogenesis [77, 82–84].

Melanin synthesis is a tightly regulated process whereby potentially cytotoxic intermediates [85] polymerize onto structural protein fibrils in melanosomes and the specialized pigmented organelle in melanocytes. Several genes involved in melanin synthesis have been implicated in iris pigment dispersion and atrophy in mice studies. *Tyrp1* encodes *tyrosinase-related protein 1*, an important melanosome membrane-bound structural component of the tyrosinase complex that oxidizes 5,6-dihydroxyindole-2-carboxylic acid (DHICA), has catalase activity, and modulates tyrosinase (*Tyr*) function [86–88]. In a screen of coat color variants, the *Tyrp1^b-lt^* (*light* coat) allele (which contains a single missense *Tyrp1* mutation) was associated with iris pigment dispersion in the LT/SvEiJ inbred mouse [82, 89]. The *Tyrp1^b^* (*brown* coat) allele has two missense mutations and has been shown to cause iris atrophy in both the DBA/2J and YBR/EiJ inbred mouse strains [77, 84, 90]. Mutation of essential cysteine residues in both *Tryp1^b-lt^* and *Tyrp1^b^* alleles causes the release of cytotoxic melanin synthesis intermediates from melanosomes leading ultimately to melanocyte cell death [87, 89]. A spontaneous coat colour variant *nm2798* is caused by the *dopachrome tautomerase* (*Dct*) allele *Dct^slt-lt3J^* and is associated with iris pigment dispersion [82]. Dct is another protein in the tyrosinase complex and also participates in melanin synthesis by converting dopachrome to DHICA [91]. Mutations of *Dct* are likely to cause melanosomal dysfunction and melanocyte toxicity via escape or accumulation of cytotoxic melanin synthesis intermediates [85, 86, 91, 92]. A large-scale genetic analysis of genetic modifiers of the iris transillumination defect in the DBA/2J mouse model identified the *oculocutaneous albinism type 2* (*Oca2*) gene, an important regulator of melanin synthesis through melanosomal pH control [93, 94]. The human homologue *OCA2* also has a direct tie to iris pigmentation, being the causative loci for both its namesake disease, oculocutaneous albinism type 2 (OMIM number 203200) and iris color [94–98].

Several genes important to melanosome function, but not involved in melanin synthesis, have also been implicated in iris pigment dispersion phenotypes. A C-terminally truncated allele of *glycoprotein nonmetastatic melanoma protein b* (*Gpnmb*) allele, *Gpnmb^r150x^*, was mapped as causing iris pigment dispersion in the DBA/2J strain [77]. Although not directly involved in melanin synthesis, *Gpnmb* is important to melanosome structural integrity and containing the cytotoxic melanin synthesis intermediates [77]. Intriguingly, C-terminal truncation of its homologue *Pmel* (*Si* allele) in mice causes melanosome dysfunction and melanocyte cell death leading to body-wide pigmentary abnormalities but not iris pigment dispersion [99–101]. *Gpnmb* has additional neuronal and immune cell adhesion functions that are important to the pathology of glaucoma in DBA/2J mice [102–104]. Understanding the immune component of *Gpnmb^r150x^*-mediated iris pigment dispersion may be important to elucidate the known involvement of the immune system in PDS [25, 105]. Similarly, *Lyst* encodes the lysosomal trafficking regulator protein which is important for trafficking components to the early stage 1 melanosomes, and variants can cause Chediak-Higashi syndrome (number 214500) [106]. The *Lyst*^*bg*-j^ allele causes *beige* coat color in the C57BL/6J background. Beige mice exhibit pronounced pigment dispersion and increased melanosome volume with similarities to both PDS and PXS [82, 83]. The underlying molecular mechanism for this phenotype is not yet understood but could be related again to cytotoxic melanin synthesis intermediates. The same large-scale genetic analysis which identified *Oca2* also identified several genes not directly involved in melanin synthesis. The motor protein *Myosin Va* (*Myo5a*) gene, signalling protein *protein kinase C ζ* (*Pkcζ*), and transcription factor *zinc finger and BTB domain-containing protein 20* (*Zbtb20*) were identified as key modifiers of the iris transillumination defect [93]. Both *Myo5a* and *Pkcζ* have a direct tie to pigmentation either through intercellular trafficking of melanosomes [107–109] or through melanocyte dendrite formation [110] (a structure important to intercellular trafficking), respectively. It is not clear how *Zbtb20* may influence this phenotype as the gene remains an understudied transcription factor with ties to the nervous, detoxification, and immune system function thus far, the latter having been already implicated in the DBA/2J mouse model previously [111–113]. Finally, in the *vitiligo* substrain of C57BL/6J mice, a variant in the master pigmented cell transcription factor *Mitf* (*Microphthalmia-associated transcription factor*) [114] caused relatively late onset pigment dispersion and increased eye size, possibly due to increased IOP^82^. The *Mitf^mi-vit^* allele likely disrupts the regulation of *Tyrp1*, *Dct*, *Gpnmb*, *Lyst*, *Myo5a*, and even *PKCζ* given *Mitf*'s essential role in regulating melanocyte identity and function [115–117].

One additional animal model exists with some relevance to PDS/PG. Canine ocular melanosis (OM) shares some phenotypic similarities with PDS/PG in that the pigment is lost from the posterior of the iris leading to transillumination defects, pigment accumulates aberrantly in the TM, and increased IOP with glaucoma developing in affected canines. However, OM is characterized by a host of other pigmentary anomalies and pathogenic phenotypes including but not limited to iris root thickening, uveal melanocytic neoplasms, large scleral/episcleral pigment plaques, fundus pigmentation, corneal edema, and anterior uveitis [118, 119]. Together, these dramatic anomalies are more reminiscent of cancer than PDS/PG making the applicability of this model to human disease limited. A genetic screen in Cairn terriers assuming an autosomal dominant mode of inheritance ruled out genes implicated in the DBA/2J model as being causative for OM [120].

Together, the body of animal research strongly supports a central role for dysregulation of melanin synthesis, melanosome integrity, and melanocyte health in the pathogenesis of PDS. Although based on the reverse genetic nature of screening coat color (and thus pigmentation) affecting variants for iris pigment dispersion, it is striking that so many different genes acting in similar processes have been associated with this phenotype in mice. In some sense, the animal literature on iris pigment dispersion is in marked contrast with human clinical research that has focused on the structural features of PDS as opposed to the cellular ones. None of the genes implicated in mouse models have yet been associated with PDS/PG in humans. While not discussed in detail in the current review, there is also a broader literature on the progression of IOP, glaucomatous optic neuropathy, axonal transport changes, and other glaucoma-associated phenotypes studied in the DBA/2J mouse model presented in detail within several informative reviews [121–123]. Interestingly, a recent large genetic analysis has revealed that the iris transillumination defect is independent of the increased IOP observed in the DBA/2J model [124]. Instead, this glaucoma-like phenotype seems to be regulated primarily by other genes such as the calcium voltage-gated channel auxiliary subunit alpha2delta1 (*Cacna2d1*) which has broader relevance for POAG [125]. Although glaucoma develops in both the DBA/2J and YBR/EiJ mouse strains, it is important to note that strong evidence suggests glaucomatous optic neuropathy may be linked to underlying neurodegeneration independent of *Tyrp1* alleles [84, 126]. This gap between the iris pigment dispersion phenotype and the glaucomatous phenotypes observed in these mouse models limits their applicability to PG but remains an interesting contrast to the research in humans.

## 6. Models of PDS/PG

**Figure 1:**
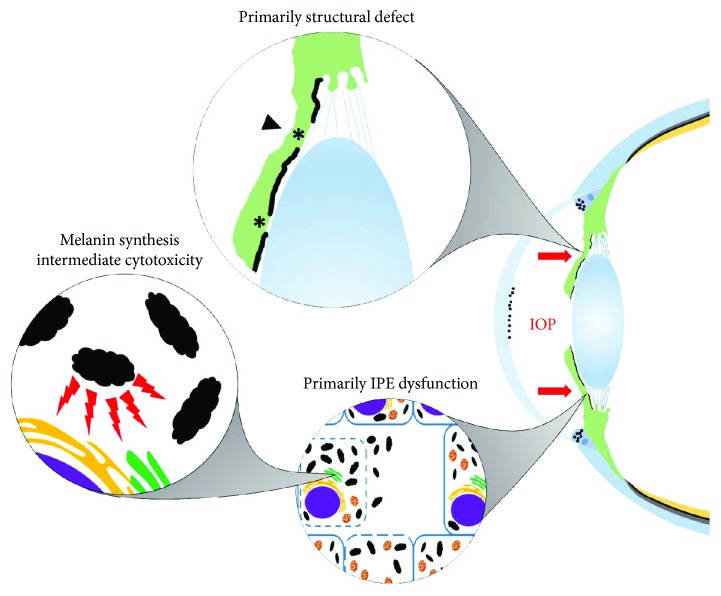
Schematic representation of PDS/PG models. In patients with PDS, pigment liberated from the posterior surface of the iris (green) circulates into the anterior chamber following the flow of aqueous humor where it deposits into the cornea and trabecular meshwork (black dots). High IOP can maintain iris bowing (red arrows) due to the reverse pupillary block in which the lens and iris act together in a ball-valve pressure system which normally acts to maintain unidirectional aqueous humor flow. There are two models of PDS/PG which differ in respect to the origin of pigment dispersion from the ciliary body to the trabecular meshwork. The *structural model* of PDS/PG proposes that posterior iris bowing creates inappropriate iridozonular contacts (black arrowhead, top circle) and that mechanical rubbing between the iris, zonules, and lens is responsible for liberating pigment from the IPE (asterisks, top circle). Although these structural features are well established, it still remains unclear if they predate pigment dispersion as the underlying mechanism. Animal models support IPE dysfunction as the primary driver of this dispersion. In this model, pigmented melanocytes die and/or detach from the IPE (bottom right circle) due to release of cytotoxic melanin synthesis intermediates from dysfunctional melanosomes (bumpy ovals, bottom left circle).

The discordance between structural features being primarily implicated in human PDS and melanocyte death being implicated in animal studies suggests it is essential to reevaluate classical models of PDS/PG. Undoubtedly, the most thoroughly investigated model of PDS/PG is the “*structural model*” in which a structural abnormality of the iris is responsible for excessive iridozonular contact which removes pigmented cells from the IPE via a mechanical rubbing force (Figure 1) [3]. Several lines of evidence support this structural model. Some patients with PDS have demonstrable iris concavity, and most are myopic further supporting some structural component [4, 7, 31, 34]. Abnormal iridozonular contact can be observed in patients with PDS where mechanical rubbing may occur [3, 4] correlating well with the midperipheral and radial distribution of iris transillumination defects [2, 22]. Mechanical strain on the eye via rigorous exercise leading to pigment liberation demonstrates the potential mechanical nature of this defect [41–44]. The concept of the reverse pupillary block acting to maintain this abnormal contact and facilitates mechanical rubbing has relevance for both PDS and pseudophakia [3, 23]. However, there are several limitations of this structural model as well. Notable is the lack of evidence supporting laser peripheral iridotomy (LPI) as a beneficial surgical intervention. LPI is designed to flatten the iris and alleviate iris concavity. Given that in a structural paradigm of PDS/PG, such an abnormality would be the source of the abnormal iridozonular contact and thus pigment shedding. However, a Cochrane review of LPI found no clear benefit of LPI in preventing loss of visual field but also rated the studies examining the technique as very low quality [127]. LPI appears to be effective at flattening the iris, thus eliminating the structural insult, but this does not prevent progression to PG [128–130]. Additionally, a paucity of evidence exists surrounding the lifespan progression PDS/PG. Structural abnormalities are associated with PDS, but whether they predate the onset of pigment shedding is unknown. It has previously been proposed that “a gene affecting some aspect of the development of the middle third of the eye early in the third trimester of fetal development may responsible for the structural defect” given the timing of iris development [22]. However, no such gene has been discovered and known anterior segment developmental control genes such as *PAX6* [131], *FOXC1* [132], and *PITX2* [133] are not associated with PDS/PG but are instead causative of other types of glaucoma [134]. It would be highly informative to carefully examine the structure of juvenile eyes, in pedigrees where PDS/PG appears to have a stronger genetic component, to address this shortfall.

An *IPE dysfunction model* of PDS/PG has the possibility to address the shortcomings of the structural model and has several interesting implications. Most notably, IPE dysfunction is best supported by the existing animal literature on iris pigment dispersion, iris atrophy, and pigmentary glaucoma. Consistently, mouse models of these phenotypes have been determined to be caused by genes controlling melanin synthesis, melanosome integrity, and melanocyte health [77, 82]. Although these models are not perfect analogues to PDS/PG, the theoretical model of IPE dysfunction provides a reasonable causal relationship between a genetic component and the observed clinical features of PDS. IPE dysfunction at the melanocyte level may be mediated by the inappropriate release of cytotoxic melanin synthesis intermediates or impaired response to cellular stresses as pigmentation is inherently a stressful process, and the iris undergoes continuous melanogenesis [85, 92, 135]. Melanocytes impaired in this way may die or detach constituting the liberated pigmented material (Figure 1). It is currently unknown if this material is comprised of melanin granules, melanosomes, or whole cellular debris, but resultant melanocyte cell death in the IPE is well established [11, 13, 25]. A melanocyte focused model has the added benefit of providing a reasonable theoretical basis for the involvement of the RPE in the pathophysiology of PDS/PG given that both structures are pigmented. The differential involvement of the tissues may be a consequence of active melanogenesis in iris melanocytes versus retinal melanocytes which seem to undergo a burst of melanosome biogenesis in development that is then retained for the patient's lifetime [135, 136]. Careful consideration thus should be given to pigmentation and/or melanocyte genes in future investigations into the genetic aetiology of PDS/PG.

## 7. Conclusion

Recent studies describing the clinical characteristics of PDS and PG and the improved understanding of the role of genes implicated in animal models of PDS/PG are calling into question classical models of the basis of this important causes of blindness. As such, we critically need new research into the fundamental basis of PDS, PG, and the relationship between these two presentations. We believe there are several important questions which researchers could investigate to better understand PDS/PG. Firstly, it would be highly informative to better describe the natural history of PDS/PG in the preclinical phase to better understand the state of the eye proceeding PDS. Large families with high incidence of PDS exist which could be worked with to address whether a structural feature of the iris proceeds PDS and what variability exists in the age of onset. Animal studies have also highlighted a vastly understudied immune component to PDS/PG. Although histologic evidence for immune involvement in PDS existed as early as 1974, human studies have not focused on this component. A better understanding if and how the immune system impacts PDS/PG onset/progression in humans as it does in DBA/2J mice may yield novel insights into the pathology of this disease. Better understanding the nature of pigment loss from the IPE may also be important to ultimately identify the underlying cause of PDS. Although unhealthy/dying melanosomes have been observed histologically, it is unknown if this cell death is the primary mode of pigment loss. As melanosomes are transferred between cells, it is possible that some pigment shedding may be due to inappropriate export of pigmented particles and that melanocyte death is a secondary phenotype. Identifying the composition of the shed pigmented material may assist in determining if shed pigment is comprised purely of melanosomes or also contain additional cell fragments. Finally, as reported conversion rates have varied greatly between ethnic groups, it is possible that these differences in genetic background may be leveraged to identify important haplotypes associated with conversion risk. Large-scale GWAS style genetic analyses may be able to identify these important risk factors and provide novel insight into genetic risks for conversion. However, this will require large-scale cohorts and rigorous phenotyping to undertake successfully. Together, answering these questions with the significant advances in genetic screening technologies and laboratory techniques will yield new insights into the genetic causes of PDS and PG, advancing our understanding of the underlying mechanisms and hopefully leading to new treatment paradigms for this common form of blindness.
